# Do scoring systems help us to estimate prognosis after mechanical thrombectomy? Data from the German Stroke Registry

**DOI:** 10.1136/jnis-2024-022772

**Published:** 2025-02-25

**Authors:** Marianne Hahn, Sonja Gröschel, Roman Paul, Luis Weitbrecht, Maria Protopapa, Sebastian Reder, Ahmed E Othman, Klaus Gröschel, Timo Uphaus, German Stroke Registry – Endovascular Treatment (GSR-ET) investigators, Arno Reich

**Affiliations:** 1 Department of Neurology, University Medical Centre of the Johannes Gutenberg University Mainz, Mainz, Germany; 2 Institute of Medical Biostatistics, Epidemiology and Informatics (IMBEI), University Medical Center of the Johannes Gutenberg University Mainz, Mainz, Germany; 3 Department of Neuroradiology, University Medical Center of the Johannes Gutenberg University Mainz, Mainz, Germany

**Keywords:** Stroke, Intervention, Thrombectomy

## Abstract

**ABSTRACT:**

**Background:**

Numerous scoring systems have been developed to individualize estimation of functional outcome after endovascular thrombectomy (EVT) of acute ischemic stroke. The aim of our study was to assess their utility for clinical practice based on a large cohort from real-world care of EVT.

**Methods:**

For 13 082 patients included in the German Stroke Registry Endovascular Treatment (GSR-ET) (July 2015 to December 2021), we calculated the following prognostic tools: pre-interventional PRE-, Totaled Health Risks in Vascular Events – Endovascular therapy (THRIVE-EVT)- and Computed Tomography for Late Endovascular Reperfusion (CLEAR) scores and post-interventional MR PREDICTS@24 hours and BET-score. Area under the receiver operating characteristic curve (AUC) analyses in the total cohort and pre-defined subgroups were performed to determine each tool’s prognostic value for good functional outcome (modified Rankin Scale (mRS) 0–2) and mortality at 90-day follow-up.

**Results:**

All pre-interventional tools achieved a moderate prognostic value for predicting good functional outcome (PRE: AUC (95% confidence interval): 0.757 (0.747–0.768), THRIVE-EVT: 0.751 (0.740–0.761), CLEAR: 0.731 (0.72–0.742)), had a higher predictive value than the admission National Institute of Health Stroke Scale ((NIHSS); 0.705 (0.694–0.716), all P<0.001), but were inferior to the NIHSS 24 hours after EVT (0.864 (0.855–0.872), all P<0.001). Predictive capacity for mortality was less accurate (AUC range: 0.697–0.729). Subgroup analyses revealed that the PRE-score was most robust at predicting good functional outcome, whereas the THRIVE-EVT score was superior in predicting mortality. Post-interventionally, MR PREDICTS@24 hours yielded high predictive accuracy for good functional outcome and mortality (both AUC >0.85), superior to 24-hour NIHSS for all subgroups, except patients <50 years of age.

**Conclusion:**

Pre-interventional scoring tools predict functional outcome after EVT better than stroke severity alone. Post-interventionally, the MR PREDICTS@24 hours tool adds predictive value to the 24-hour NIHSS as a single prognostic feature. Multivariate prognostic tools incorporating (post-)procedural information enable individualization of prognosis assessment after EVT under routine-care conditions.

**WHAT IS ALREADY KNOWN ON THIS TOPIC**

- Numerous scoring tools for outcome prognostication in patients with acute stroke have been developed to aid clinical decision making. Little is known about their predictive accuracy in large cohorts of endovascular thrombectomy (EVT) in clinical practice.

**WHAT THIS STUDY ADDS**

- We provide evidence from a large, multicenter, prospective registry study of EVT under routine-care conditions, suggesting that pre-interventional scoring tools are superior to stroke severity to estimate prognosis in EVT-treated patients. Good functional outcome was most accurately predicted by the Pittsburgh Response to Endovascular therapy score (PRE), while the Totaled Health Risks in Vascular Events score – Endovascular therapy (THRIVE-EVT) was superior at predicting mortality. Post-interventionally, the MR PREDICTS@24 hours provides high prognostic value for both good outcome and mortality, significantly more accurate than outcome prognostication by the National Institute of Health Stroke Scale ((NIHSS) 24 hours after EVT alone.

**HOW THIS STUDY MIGHT AFFECT RESEARCH, PRACTICE OR POLICY**

- Multivariate prognostic tools incorporating (post-)procedural information enable individualization of prognosis assessment after EVT under routine-care conditions. Since EVT is increasingly performed also in patients suspected to have high chances for severe persisting deficits (large core, late time window, etc.), post-interventional tools for prognosis estimation may become increasingly relevant for clinical decision making and navigating patient pathways in the future.

## Introduction

 Endovascular thrombectomy (EVT) is a highly effective treatment in large vessel occlusion (LVO) ischemic stroke, which has become standard of care in acute stroke treatment. Prognosis estimation after EVT of LVO is difficult due to various influencing factors, yet, essential for individualized decision making throughout the pathway of acute stroke care. Therefore, several scoring systems for patient selection and prognostication of long-term outcomes following EVT of LVO ischemic stroke have been developed.^1^ They are meant to support clinical decision making throughout the course of acute stroke care by either assessing the probability of benefit from EVT before the intervention or by estimating the odds for good or poor long-term outcome after the acute stroke treatment.

Features included in the scoring systems vary over the different tools. Most pre-interventional prognostic tools rely on a patient’s age and stroke severity, measured by admission National Institute of Health Stroke Scale (NIHSS).^2^,^7^ Some also consider cerebrovascular risk factors, such as arterial hypertension, diabetes mellitus, and atrial fibrillation.^2^ Others include imaging features such as the Alberta Stroke Programme Early CT Score (ASPECTS)^2^,^5^ or advanced imaging parameters such as cerebral blood volume index (CBV).^7^ Laboratory parameters, such as serum glucose measures, are also contained in some scoring instruments.^5^,^7^ For post-EVT prognosis estimation, including procedural or post-procedural information, only a few tools exist,^8^,^13^ most of them developed in small patient cohorts. Among included procedural and post-procedural features are recanalization status, intravenous thrombolysis (IVT), general anesthesia used during EVT, time to flow restoration, final infarct volume, and post-interventional intracranial hemorrhage, depending on the instrument.

Validation of scoring tools for outcome prognostication after EVT in contemporary datasets and routine care cohorts of EVT is scarce but highly necessary to assess their potential to guide clinical decisions for individual patients. Within this study, we estimate and compare the performance of previously published pre- and post-EVT prognostic scores predicting functional outcome after EVT in a large, prospective, multicenter, observational dataset of EVT under routine care conditions. We investigate the added value of scoring tools compared with single feature prognostic measures by comparing them to admission NIHSS and NIHSS 24 hours after EVT. Secondly, we compare accuracy of prediction over different subgroups of patients in order to evaluate the robustness of predictive accuracy in a heterogeneous cohort and analyze the prognostic value of scoring tools at the time point of discharge from the treating hospital.

## Methods

### Standard protocol approval

Study protocols and procedures were conducted in compliance with the Declaration of Helsinki. The German Stroke Registry – Endovascular Treatment (GSR-ET) is registered at ClinicalTrials.gov (Identifier: NCT03356392) and was approved by the ethics committee of the leading center (Ludwig-Maximilians University Munich, protocol number: 689–15), and by the local ethics committees.

### Study population

**Figure 1:**
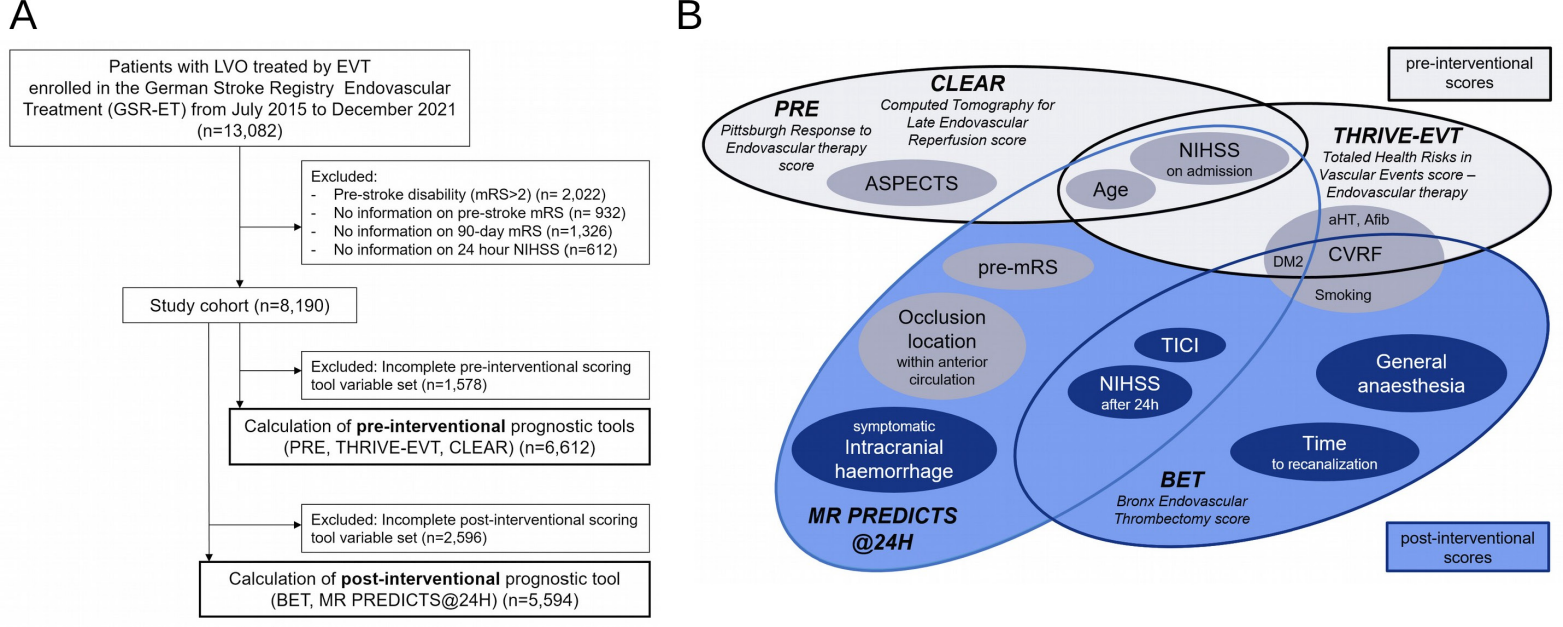
(**A**) Derivation of study cohort. (**B**) Parameters used in the investigated scoring tools. Afib, atrial fibrillation; aHT, arterial hypertension; ASPECTS, Alberta Stroke Programme Early CT Score; BET, Bronx Endovascular Thrombectomy score; CLEAR, Computed Tomography for Late Endovascular Reperfusion score; CVRF, cerebrovascular risk factors; DM2, diabetes mellitus; EVT, endovascular treatment; LVO, large vessel occlusion; mRS, modified Rankin scale score; NIHSS, National Institute of Health Stroke Scale; PRE, Pittsburgh Response to Endovascular therapy score; THRIVE-EVT, Totaled Health Risks in Vascular Events score – Endovascular therapy; TICI, trombolysis in cerebral infarction scale score.

The GSR-ET is an ongoing academic, independent, prospective, multicenter, observational registry study. Twenty-five certified German stroke centers consecutively enroll adult patients diagnosed with acute ischemic stroke due to LVO and intention to treat with EVT. Baseline demographics, comorbidities, clinical and procedural information as well as clinical follow-up after 90 days are recorded. More detailed information on the registry’s study protocol and variables has been published before.^14^ Of the 13 082 patients enrolled in the GSR-ET from July 2015 to December 2021, we excluded patients with significant pre-stroke disability (modified Rankin Scale (mRS)>2) (n=2022), as well as patients with no information on pre-stroke mRS (n=932), 90-day mRS (n=1326), or 24-hour NIHSS (n=612). In the resulting study cohort of n=8190 patients, patients who were missing information on variables needed for calculation of pre-interventional scoring tools (n=1578) or for post-interventional tools (n=2596) were excluded for the respective primary analysis (figure 1, for baseline characteristics of study cohort, see table 1).

**Table 1:** Baseline characteristics of the study cohort

Variable	Number of patients (percentage) / Median (IQR) (number of patients)
N	8190
Age	75 (64-82) (n=8185)
Female sex	49.0% (4,014/8,186)
Cardiovascular risk factors	
Arterial hypertension	75.9% (6,192/8,160)
Diabetes mellitus	21.4% (1,749/8,160)
Dyslipidemia	41.2% (3,359/8,147)
Atrial fibrillation	39.3% (3,206/8,154)
Smoker (current)	17.2% (1,328/7,709)
Baseline medication	
Anticoagulation	23.0% (1,867/8,103)
Platelet inhibition	29.3% (2,373/8,103)
Stroke severity	
NIHSS on admission	14 (8-18)(n=8117)
Location of occlusion	
Carotid artery	26.2% (2,109/8,057)
ACA	2.8% (227/8,057)
MCA M1	51.4% (4,145/8,057)
MCA M2	22.7% (1,829/8,057)
PCA	2.9% (240/8,057)
VB	9.9% (797/8,057)
Stroke etiology	
Large artery atherosclerosis	25.7% (2,084/8,121)
Cardioembolism	49.5% (4,023/8,121)
Dissection	2.0% (166/8,121)
Other	4.8% (386/8,121)
Undetermined	18.0% (1,462/8,121)
Treatment characteristics	
Intravenous thrombolysis	49.7% (4,054/8,164)
Primary admission at the MT site	59.1% (4,670/7,903)
Symptom onset/time of recognition-to-admission (minutes)	112 (60-200) (n=7409)
Door-to-groin puncture (minutes)	70 (47-100) (n=7758)
Outcome parameters	
Successful reperfusion (TICI 2b-3)	86.0% (6,895/8,016)
Duration of hospital stay (days)	9 (5-13) (n=8165)
Good outcome at discharge (mRS 0–2)	33.3% (2,584/7,766)
In-house mortality (mRS 6)	17.5% (1,360/7,766)
Good outcome at 90-day follow-up (mRS 0–2)	41.0% (3,354/8,190)
Mortality (mRS 6) at 90-day follow-up	25.7% (2,102/8,190)

Data are presented as percentage (absolute number) except for age, NIHSS on admission, procedure times and duration of hospital stay: median (IQR).

ACA, Anterior cerebral artery; MCA M1, Middle cerebral artery M1 segment; MCA M2, Middle cerebral artery M2 segment; mRS, modified Rankin Scale; NIHSS, National Institutes of Health Stroke Scale; PCA, Posterior cerebral artery; TICI, Thrombolysis in cerebral infarction scale score; VB, vertebrobasilar.

### Calculation of prognostic scoring tools

We reviewed the literature for published multiparametric scoring tools for good/bad functional outcome after EVT containing pre-interventional and/or post-interventional parameters. The investigated scores and calculations are displayed in table 2. Tools including parameters that were not available within the GSR-ET database (eg, advanced imaging parameters, laboratory parameters) could not be calculated and were therefore not analyzed (see online supplemental table S1).

**Table 2:** Scores for prognosis estimation in patients with LVO strokes, calculated according to the original publications

Predictive instrument	Scoring rule
Pre-interventional prognosis estimation	
Pittsburgh Response to Endovascular therapy (PRE)-score^3^	= age + (2 x admission NIHSS) – (10 x ASPECTS)
Totaled Health Risks in Vascular Events (THRIVE-EVT)-score^2^	= 2.864 + (−0.025 x age) + (−0.106 x admission NIHSS) + X + 0.753, Where X is determined by the number of risk factors present out of arterial hypertension, diabetes mellitus, atrial fibrillation: X=−0.419 (one risk factor) or −0.502 (two risk factors) or −1.665 (three risk factors).
Computed Tomography for Late Endovascular Reperfusion (CLEAR -score^4^	= age (<51 years: 2 points, 51–75 years: 1 point, >75 years: 0 points) + ASPECTS (>5: 1 point, 0–5: 0 points) + admission NIHSS (<6: 5 points, 6–12: 3 points, 13–18: 1 point, >18: 0 points).
Post-interventional prognosis estimation	
Bronx Endovascular Thrombectomy (BET)-score^8^	= current smoker, diabetic, general anesthesia, time from puncture to recanalization, TICI <3: each one point, post-EVT NIHSS >9: two points.
MR PREDICTS@24 hours prediction tool^11^	Calculated according to the probability function provided in the original publication (including: age, admission NIHSS, diabetes mellitus, premorbid mRS, occlusion location within anterior circulation, TICI, NIHSS 24-hours after EVT, symptomatic intracranial hemorrhage).

ASPECTS, Alberta Stroke Programme Early CT Score; EVT, endovascular therapy; NIHSS, National Institutes of Health Stroke Scale; TICI, Thrombolysis in cerebral infarction scale score.

### Statistical analysis

For estimation of prognostic accuracy, area under the receiver operating characteristic curve (AUC) comparisons were performed. The primary outcome parameter was good functional outcome (mRS 0–2) at 90-day follow-up and 90-day mortality was analyzed as a secondary outcome. Two separate AUC-analyses to assess the prognostic value of scoring tools containing pre-interventional information and those containing post-interventional information were carried out.

AUC of scoring tools containing pre-interventional information (Pittsburgh Response to Endovascular therapy score (PRE), Totaled Health Risks in Vascular Events score – Endovascular therapy (THRIVE-EVT), and Computed Tomography for Late Endovascular Reperfusion score (CLEAR), were compared with AUC of admission NIHSS and NIHSS 24 hours after EVT (24H-NIHSS). Similarly, AUC of the scoring tools also containing procedural and post-procedural information (Bronx Endovascular Thrombectomy score (BET), MR PREDICTS@24 hours) was compared with AUC of 24H-NIHSS. Furthermore, model calibration was performed to assess the models’ goodness of fit. To assess the consistency of predictive accuracy, subgroup AUC comparison analyses of the scoring systems were carried out over the following groups: age (<50 years, 50–75 years, >75 years), sex, time window (last seen well/symptom onset to admission <vs> 6 hours), stroke severity (admission NIHSS <10, 10–19, >19), ASPECTS (0–5 versus>5), IVT (bridging IVT vs EVT alone), and recanalization status (trombolysis in cerebral infarcation scale score (TICI) 0 to 2a vs 2b-3). To assess predictive capacity of prognostic scoring systems at the time point of discharge from the treating hospital, a subgroup analysis was carried out in the population of patients discharged alive from the treating hospital. Youden index calculation was performed to assess optimal criterion value, sensitivity, and specificity for prognosis estimation. Since the primary analysis was conducted on a complete cases dataset, a sensitivity analysis using multiple imputation was performed to assess potential selection biases. Additionally, a sensitivity analysis was conducted in patients with pre-stroke mRS>2. All p-values are considered exploratory are performed on retrospective data, thus no adjustment for multiplicity was performed; p-values of <0.05 are considered statistically significant. Statistical analyses were performed using SPSS (Version 29, IBM, Armonk, NY, USA) and SAS (Version 9.4, SAS Institute, Cary, NC, USA).

## Results

### Predictive accuracy of pre-interventional prognostic scores

#### Good functional outcome

**Figure 2:**
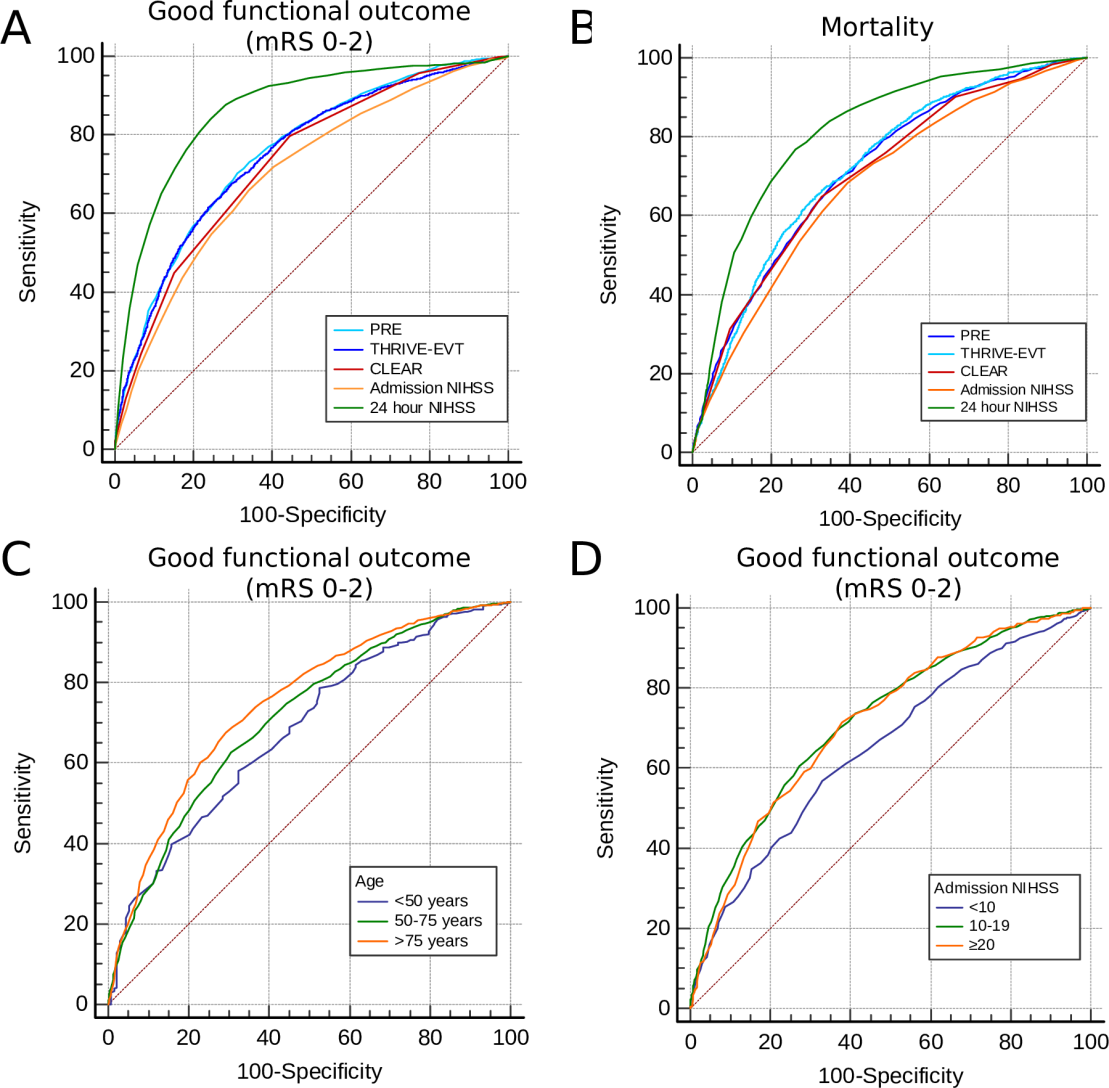
Predictive accuracy of pre-interventional scores. A+B: AUC-comparison of pre-interventional prognostic tools with admission NIHSS and NIHSS 24 hours after EVT predicting good functional outcome (**A**) and mortality (**B**) 90 days after EVT. C+D: Comparison of independent ROC-Curves comparing predictive accuracy of PRE-score depending on age (**C**) and stroke severity (**D**). PRE, Pittsburgh Response to Endovascular therapy score; THRIVE-EVT, Totaled Health Risks in Vascular Events score – Endovascular therapy; CLEAR, Computed Tomography for Late Endovascular Reperfusion score.

We report moderate accuracy of all three predictive tools (PRE, THRIVE-EVT, CLEAR) in prediction of good functional outcome 90 days after EVT, depicted by AUCs all greater than 0.7 (table 3, figure 2A). Although all predictive tools were significantly superior to the admission NIHSS as single prognostic feature (all p<0.001) in AUC comparison, PRE (AUC (95% CI): 0.757 (0.747–0.768)) and THRIVE-EVT (0.751 (0.740–0.761)) were significantly superior to CLEAR (0.731 (0.720–0.742), all p<0.001). AUC superiority to admission NIHSS persisted only for PRE in all pre-defined subgroups (online supplemental table S2). In independent AUC comparison of subgroups, PRE revealed significantly better predictive capacity in patients aged >75 years, females, admission NIHSS >10 and ASPECTS 0–5 (figure 2C+D). In the late time window (>6 hours after symptom onset or time of recognition), for which CLEAR was initially developed, the CLEAR-score was equally accurate as PRE, yet not superior.

**Table 3:** Comparative AUC analyses of LVO outcome prediction by pre-interventional scoring tools and NIHSS

Predictive instrument	AUC	95% CI
Outcome parameter: Good functional outcome (mRS 0–2) at 90-day follow-up		
Admission NIHSS	0.705	0.694 to 0.716
PRE	0.757*	0.747 to 0.768
THRIVE-EVT	0.751*	0.740 to 0.761
CLEAR	0.731	0.720 to 0.742
NIHSS 24 hours after EVT	**0.864†**	0.855 to 0.872
Outcome parameter: 90-day mortality		
Admission NIHSS	0.682	0.671 to 0.693
PRE	0.717*	0.706 to 0.728
THRIVE-EVT	0.722*	0.712 to 0.733
CLEAR	0.705	0.694 to 0.716
NIHSS 24 hours after EVT	**0.814†**	0.805 to 0.824

* significantly higher AUC than non-bold scoring tools.

† significantly higher AUC than all other scoring tools.

AUC, area under the receiver operating characteristic curve; CLEAR, Computed Tomography for Late Endovascular Reperfusion score; EVT, endovascular therapy; NIHSS, National Institutes of Health Stroke Scale; PRE, Pittsburgh Response to Endovascular therapy score; THRIVE-EVT, Totaled Health Risks in Vascular Events score – Endovascular therapy.

#### Mortality

Prediction of 90-day mortality by all three predictive tools was less accurate than prediction of good functional outcome, with still moderate predictive capacity overall (all AUCs >0.7, table 3, figure 2B). Also for mortality, all scoring instruments were superior to the admission NIHSS, while PRE (AUC: 0.717 (0.706–0.728)) and THRIVE-EVT (0.722 (0.712–0.733) were significantly superior to CLEAR. AUC superiority to admission NIHSS persisted only for THRIVE-EVT in all pre-defined subgroups (online supplemental table S2). In independent AUC comparison of subgroups, THRIVE-EVT revealed significantly better predictive capacity in patients aged <50 years, in the late time window, with admission NIHSS <20 and bridging IVT. In the late time window, CLEAR-score was equally accurate as THRIVE-EVT, but again, not superior.

#### Predictive accuracy of pre-interventional prognostic scores compared with NIHSS 24 hours after EVT

**Figure 3:**
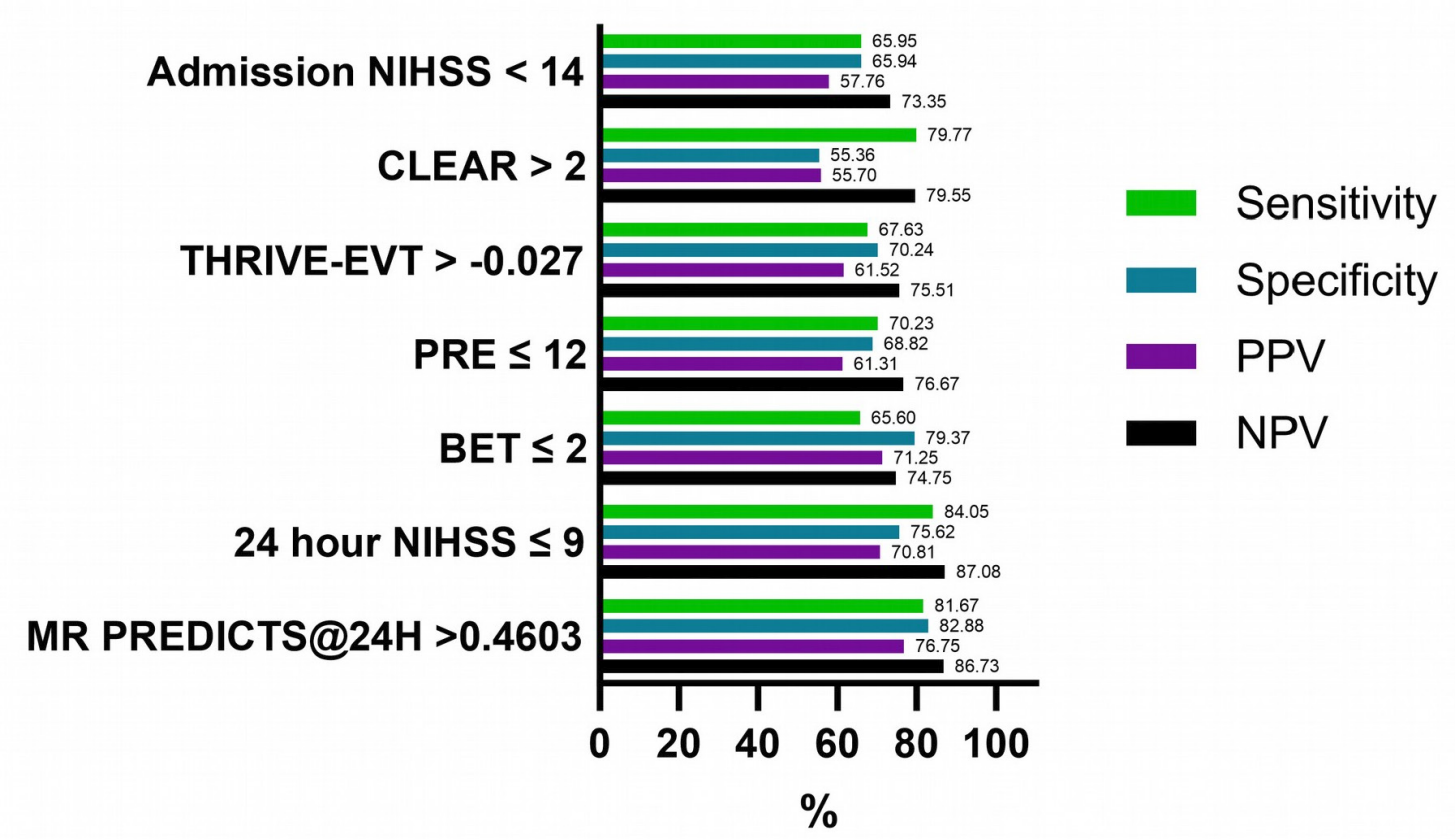
Sensitivity, Specificity, positive predictive value (PPV) and negative predictive value (NPV) of scoring instruments and optimal criterion value of NIHSS for good functional outcome (mRS 0–2) at 90-day follow-up. NIHSS, National Institute of Health Stroke Scale; PRE, Pittsburgh Response to Endovascular therapy score, THRIVE-EVT, Totaled Health Risks in Vascular Events score – Endovascular therapy; CLEAR, Computed T for Late Endovascular Reperfusion score; BET, Bronx Endovascular Thrombectomy score.

All predictive scoring tools were inferior to 24H-NIHSS in prediction of good functional outcome and mortality (AUC for good functional outcome: 0.864 (0.855–0.872), sensitivity: 84.05%, specificity 75.62% for NIHSS ≤9, table 3, figure 3). Of note, in the subgroup of patients with ASPECTS ≤5, the pre-interventional scoring instruments were not inferior to 24-hour NIHSS in prediction of 90-day mortality (online supplemental table S2).

### Predictive accuracy of post-interventional prognostic scores

**Figure 4:**
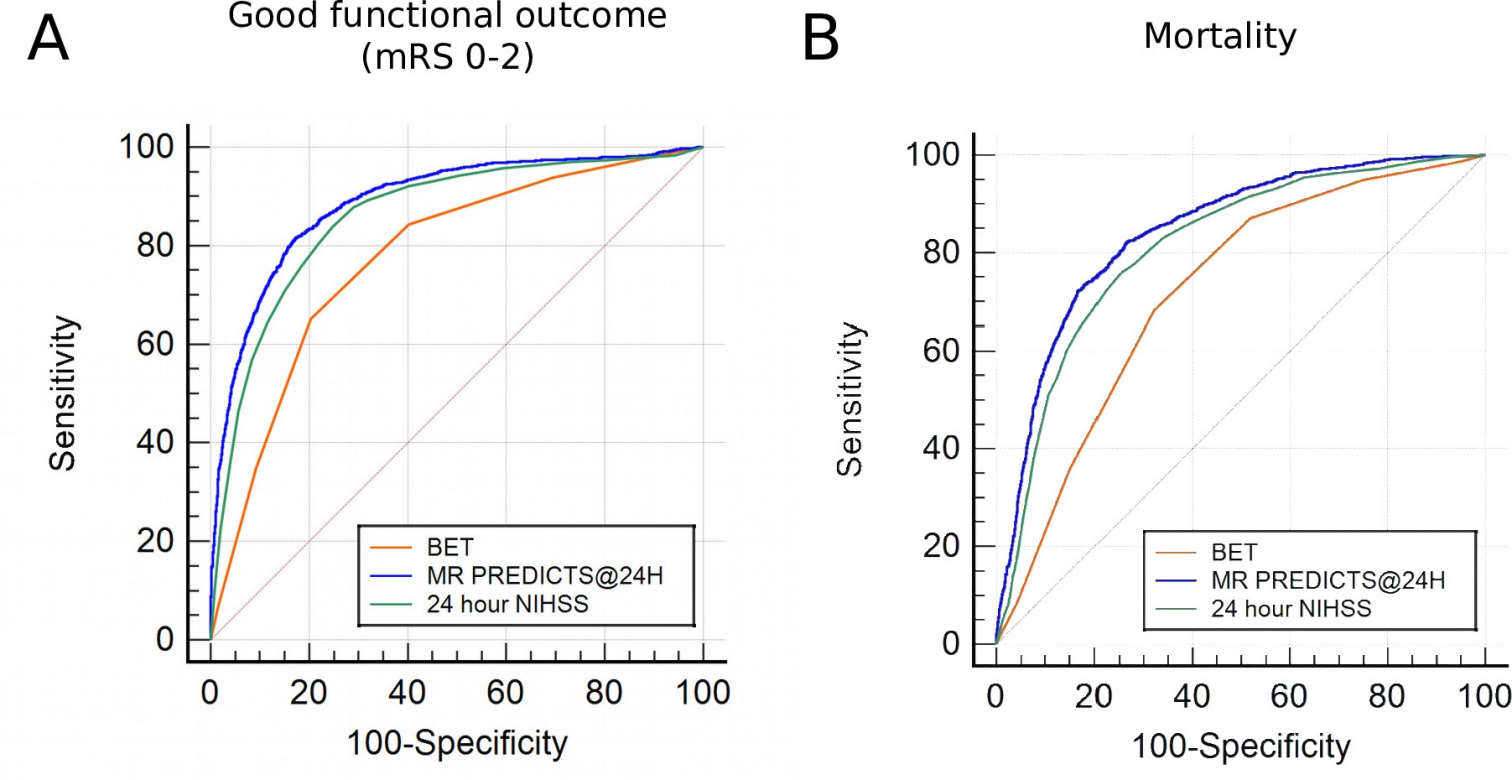
Predictive accuracy of post-interventional scores. AUC comparison of post-interventional prognostic tools with NIHSS 24 hours after EVT predicting good functional outcome (**A**) and mortality (**B**) 90 days after EVT. BET, Bronx Endovascular Thrombectomy score.

We report only moderate predictive accuracy of the post-interventional BET-score on both good functional outcome and mortality (AUC >0.7). The MR PREDICTS@24 hours tool yielded high predictive accuracy for both outcomes (good functional outcome: 0.889 (0.881–897), mortality: 0.843 (0.834–0.853), table 4, figure 4). The 24H-NIHSS was significantly superior to BET with an AUC >0.8 (all P<0.001), yet inferior to MR PREDICTS@24 hours. High predictive capacity of good functional outcome (AUC >0.85) by MR PREDICTS@24 hours persisted over all pre-defined subgroups (online supplemental table S2), except for admission NIHSS <10, where prognostic accuracy was significantly inferior compared with higher stroke severity. For young patients <50 years of age, 24H-NIHSS was as accurate as MR PREDICTS@24 hours in predicting good functional outcome. Analysis of model calibration showed that all tools are at least reasonably calibrated regarding their ability to predict good outcome, whereas with the 24H-NIHSS, potentially non-linear adjustment may be necessary (online supplemental figures S2 and S3).

**Table 4:** Comparative AUC analyses of LVO outcome prediction by post-interventional scoring tools and NIHSS

Predictive instrument	AUC	95% CI
Outcome parameter: Good functional outcome (mRS 0–2) at 90-day follow-up		
BET	0.777	0.766 to 0.788
MR PREDICTS@24 hours	0.889*	0.881 to 0.897
NIHSS 24 hours after EVT	0.861	0.851 to 0.870
Outcome parameter: 90-day mortality		
BET	0.721	0.709 to 0.733
MR PREDICTS@24 hours	0.843*	0.834 to 0.853
NIHSS 24 hours after EVT	0.813	0.802 to 0.823

* significantly higher AUC than non-bold scoring tools.

AUC, Area under the receiver operating characteristic curve; BET, Bronx Endovascular Thrombectomy score; EVT, endovascular therapy; NIHSS, National Institutes of Health Stroke Scale.

### Sensitivity analyses

In patients with pre-stroke mRS>2, all pre-interventional scoring tools resulted in AUCs <0.7 for prognostication of return to pre-stroke mRS and mortality. Likewise, AUCs of post-interventional scores were reduced in patients with pre-stroke mRS >2, compared with the primary analysis, yet the MRPREDICTS@24** **hours still yielded an AUC >0.8 for prediction of desirable functional outcome and mortality (see also online supplemental tables S3 and S4). Multiple imputation of score parameters and functional outcome did not show relevant differences in prognostic accuracy of pre- and postinterventional tools compared with complete case analysis for the total study cohort and the subgroup of patients with pre-stroke disability (for details, see online supplemental tables S3–S5).

### Prognosis estimation in patients discharged from treating hospital

A subgroup analysis was conducted to estimate predictive accuracy of scoring tools with regard to 90-day outcome at the time point of discharge from the treating hospital. In patients discharged alive from the treating hospital, PRE and THRIVE-EVT still yielded moderate predictive accuracy (AUCs >0.7, online supplemental table S6 and figure S1A) in predicting good functional outcome, although reduced compared with the total pre-interventional cohort and inferior compared with 24H-NIHSS. Accuracy of mortality prediction was clearly diminished in both scores (PRE: 0.685 (0.672–0.697), THRIVE-EVT 0.691 (0.678–0.703)), although 24H-NIHSS was also not superior to scoring tools (0.706 (0.693–0.718), all P>0.05, online supplemental table S6 and figure S1B). Of both post-interventional scores, BET yielded similar results to pre-interventional scores with moderate predictive accuracy of good functional outcome and clearly reduced mortality prognostication (0.645 (0.633–0.660)). MR PREDICTS@24 hours still yielded high predictive accuracy for good functional outcome in patients discharged from hospital (0.859 (849–0.869)), while predictive accuracy of 90-day mortality was only moderate (0.760 (0.748–0.773), online supplemental table S7 and figure S1C and D). Both outcomes were more accurately predicted by MR PREDICTS@24 hours than by 24H-NIHSS.

## Discussion

We here provide data on predictive performance of scoring tools to estimate functional outcome after EVT of LVO in a large, prospective, multicenter registry study of routine-care EVT. We show that pre-interventional scoring tools provide an additional benefit compared with admission NIHSS in prognostication of good functional outcome 90 days after EVT. Specifically, the PRE-score yielded consistent moderate predictive accuracy over all pre-defined subgroups in our cohort of EVT-treated patients, superior or equal to the other investigated tools. Therefore, we suggest the PRE-score (including age, admission NIHSS and ASPECTS) as the most robust pre-interventional scoring tool for prediction of good functional outcome after EVT. All pre-interventional scoring instruments were inferior to the 24H-NIHSS as a single prognostic feature, which was also the case for the post-interventional BET-score. This is supported by various analyses illustrating a strong predictive power of 24H-NIHSS for long-term functional outcome in EVT-treated patients.^15^ By contrast, the multivariate post-interventional MR PREDICTS@24 hours prognostic tool yielded high predictive accuracy of good functional outcome in our dataset, significantly superior to 24H-NIHSS, suggesting a potential added benefit for outcome prognostication in clinical practice. However, none of the tested instruments reached excellent measures of discriminative ability, emphasizing that outcome prognosis in an individual patient comprises a relevant share of uncertainty, also in multiparametric approaches.

Prognostication of mortality with scoring tools was less accurate than for good functional outcome. This may result from the fact that most EVT-prognostic scoring tools do not account for comorbidities contributing to non-stroke-associated death in this cohort. Moreover, only some of the scoring tools target mortality as an outcome variable in the derivation process. Interestingly, we report that THRIVE-EVT is the most valuable pre-interventional scoring tool for prognostication of 90-day mortality in our cohort, based on predictive accuracy and consistency of superiority over subgroups. In line with the previously suspected role of comorbidities in mortality prediction, THRIVE-EVT is the only pre-interventional score containing cerebrovascular risk factors, which might explain its superiority. Future development of predictive instruments may therefore benefit from assessing different parameter sets for prediction of mortality and for good functional outcome.

Derivation of scoring tools for outcome prediction after EVT was conducted in datasets with different inclusion criteria of EVT-treated patients, regarding for example, time window, vessel territories and premorbid disability. Therefore, also scoring tools for previously underrepresented patients in derivation cohorts were developed. Among them is the CLEAR score, which was derived from patients treated later than 6 hours after symptom onset. We report that, in this specific subgroup of patients in the late time window, the CLEAR-score is not inferior to PRE and THRIVE-EVT in prediction of good functional outcome and mortality, yet also not superior, depicting the challenge to augment the moderate accuracy of pre-interventional outcome prognostication by homogenization of the cohort. Supporting this, we did not observe any significant difference in PRE-score predictive accuracy of good functional outcome in our subgroup analysis of early vs late time window that would justify a subgroup-specific scoring instrument depending on time window, which may render application in clinical practice impracticable.

However, we report significant differences in predictive capacity of good functional outcome with regard to age, sex, stroke severity and ASPECTS. It may be promising therefore, to investigate whether a scoring system for young patients (<50 years of age) and patients with lower stroke severity (admission NIHSS <10), both in which moderate predictive accuracy could not be reached by PRE-score, may add value by reducing prognostic uncertainty following EVT in this subgroup of patients. Especially patients with lower stroke severity may benefit from multiparametric tools for outcome prognostication, since our data show that 24H-NIHSS performs poorly in this subgroup.

Notably, in patients with ASPECTS ≤5, the pre-interventional scoring instruments were not inferior to 24-hour NIHSS in prediction of 90-day mortality. We hypothesize that this is due to low rates of early neurologic improvement in the group of patients with low ASPECTS, as was reported recently with only 11.5% in the SELECT2 RCT for patients with ASPECTS 3-5 (16). Delayed neurological recovery may therefore impair the prognostic value of 24H-NIHSS for 90-day outcome in these patients. Consequently, the time point 24 hours after EVT may not be as valuable for prognostication of 90-day functional outcome in patients with low ASPECTS as compared with higher ASPECTS. Future studies should therefore investigate differences in the optimal time point of prognostication after EVT in high vs low ASPECTS-patients, to enable individual prognosis estimation as a basis for clinical decision making in this cohort of patients with worse prognosis due to the nature of their disease.

Scoring tools for outcome prediction following EVT were initially developed to support patient selection for EVT and therefore to be applied before acute stroke treatment. Taking into account recent evidence, suggesting therapeutic benefit of EVT also in patients with a high chance of severe persisting deficits, e.g., patients with large infarct core may be treated with EVT more often in the near future.^16^,^18^ It may therefore become less important in clinical decision making for or against EVT to identify patients with the highest odds for a good outcome. However, individualized prognostication of functional outcome will become more relevant at the time point after EVT in order to guide conversations between patient, physician and relatives and in order to set goals of care and discuss possible patient pathways after the acute stroke treatment. A later time point for outcome prognosis in acute stroke care enables inclusion of procedural and early post-procedural outcome parameters in predictive tools enhancing certainty of prediction. Few post-interventional tools have been developed, of which the tested BET-score does not yield predictive capacity superior to 24H-NIHSS in our cohort of EVT-treated patients in routine care. The MR PREDICTS@24 hours, however, an online tool for outcome prognostication 24 hours after EVT of anterior circulation LVO, which is based on a regression-derived probability distribution function for different states of functional outcome, yielded high predictive capacity, significantly superior to 24H-NIHSS for both good functional outcome and mortality. Our analysis therefore suggests a clinical benefit for outcome prognostication by MR PREDICTS@24 hours compared with neurological impairment (24-hour NIHSS) alone.

High accuracy is necessary for clinicians to trust predictive models for clinical decisions and in patient-relative-doctor communication. Although simplified sum scores, mostly including categorical variables, are easy to use, they may not add benefit to clinical practice, given only a small increase in predictive power over the 24H-NIHSS alone. Increasing model complexity might be necessary to depict the variability of outcomes and its numerous predictors in LVO and increase meaningfulness of individual outcome prediction, yet, may come along with less viability in clinical situations. Given technological progress and increasing acceptance of online resources to support clinical work, online calculators (as MR PREDICTS@24 hours is one, for example) that process easily accessible input parameters in complex probabilistic functions could become a non-inferior medium in many clinical settings.

We observed reduced predictive accuracy of pre- and post-interventional scoring tools as well as 24H-NIHSS in the subgroup of patients discharged from the treating hospital. Predictive accuracy of PRE-score and THRIVE-EVT was still moderate for good functional outcome (high for MR PREDICTS@24 hours) but clearly diminished for predicting 90-day mortality after discharge from hospital. This is especially noteworthy, since the risk of mortality might be an important argument in post-acute clinical decision making within the care pathway of patients with LVO. We hypothesize that diminished predictive accuracy of 90-day mortality in discharged patients is due to a significant difference between predictors of early mortality^19^ and predictors of mortality after discharge, especially in patients with LVO. Future studies should therefore investigate scoring systems for post-EVT outcome prognostication in more detail, aggregating prognostic features for individual outcome prognostication after EVT throughout the hospital stay.

Our study approach had several limitations. Due to the study design being a retrospective analysis of prospectively collected observational data, validation of our findings in prospective analyses is necessary. We were able to compare predictive accuracy of several scoring instruments for good functional outcome and test consistency of findings over clinically relevant subgroups, yet, further predictive tools developed for mortality prediction exist. Due to the nature of our dataset, some scoring tools for good functional outcome could not be calculated due to absence of necessary parameters.^20^ Among them are pre-interventional instruments that include advanced imaging features and laboratory parameters^5^,^7^ and post-interventional tools containing final infarct volume and complications.^10 12 13^ At the same time, included variables in our dataset are comparatively easily assessable and therefore practical to apply in outcome prognostication in clinical practice. Furthermore, inclusion criteria and populations from which the examined scores were derived are not perfectly congruent. Derivation cohorts are differing with regard to time window and vessel territories, and differences in study cohorts were described to drive differences in outcome prediction accuracy before.^21^ We hypothesize that assessing the validity of the tested scores in a population with the exact inclusion criteria the scoring tool was derived from, may result in higher accuracy of outcome prognostication, although the predictive accuracies we observed in our study ranged not far from the original publications. At the same time, we believe that a scoring system that enables outcome prognostications with adequate validity and applicable to the broad range of patients treated with EVT may be favorable in clinical practice. Our study is also limited with regard to transferability of results to patients with premorbid disability, since we excluded patients with premorbid mRS >2 from our analyses in order to reduce heterogeneity. A sensitivity analysis within this group of patients confirmed diminished prognostic value of the tested tools in patients with significant premorbid disability. This points out the need for future studies providing tools for individual outcome prognosis before and after ETV in patients with significant pre-stroke disability.

By analyzing an up-to-date large and complex, nationwide prospective cohort of >13 000 routine-care EVT procedures, our study benefits from a strong data foundation. We believe that especially comparing predictive accuracy of scoring systems with single-feature prognostic outcome parameters, such as the 24H-NIHSS is of value to assess added benefit. Furthermore, our dataset enabled us to investigate the prognostic value of the tested tools at different time points within the course of acute stroke care. This is especially important, since outcome prognostication is not solely relevant (and may be of even less importance in the future) in the pre-EVT setting but may be increasingly needed in the post-acute phase after acute treatment. Therefore, future studies need to distinguish meaningfulness of prognostic features in the acute pre-interventional setting and at later time points such as the day after EVT or on discharge from the treating hospital.

## Conclusion

Pre-interventional scoring tools for individualized outcome prognosis after EVT were found to add benefit in predictive accuracy compared with admission NIHSS alone, yet are inferior to prognosis by 24H-NIHSS. For good functional outcome, especially the PRE-score was superior to other scoring instruments consistently over subgroups. For prediction of 90-day mortality the THRIVE-EVT score seems to be the most robust instrument among those tested. Some differences in prognostic accuracy of good functional outcome were found over subgroups, for example, younger age and lower stroke severity, which may imply diminished explanatory power of pre-interventional scoring systems in these groups. Of the tested post-interventional scoring tools, only the MR PREDICTS@24 hours yielded high predictive accuracy, superior to post-EVT neurological impairment, measured by 24H-NIHSS, suggesting a potential benefit for outcome prognostication in clinical practice. Prognostic accuracy of all tools was diminished in the subgroup of patients discharged alive from the treating hospital especially with prognostication of 90-day mortality in this subgroup not reaching high accuracy with multiparametric tools nor 24H-NIHSS. Individualized outcome prognostication to support clinical decision making may become even more important in the post-acute phase after EVT, since recent evidence suggests therapeutic benefit of EVT even in patients with high odds of severe persisting deficits, such as low ASPECT scores. Therefore, future studies need to address scoring systems for individualized outcome prediction for application throughout the pathway of stroke care after EVT in more detail.

## Supplementary material

10.1136/jnis-2024-022772 online supplemental file 1

## Data Availability

Data are available upon reasonable request.
