# Supplementary material for: Do scoring systems help us to estimate prognosis after mechanical thrombectomy? Data from the German Stroke Registry
Source: J Neurointerv Surg. 2025 Feb 25;18(2):e022772. doi: 10.1136/jnis-2024-022772 (PMC12911615; doi:10.1136/jnis-2024-022772)
Supplement: online supplemental file 1 [file jnis-18-2-s001.pdf]

Table S1: Prognostic scoring tools that were identified but not included in our comparative analysis.

**Pre-interventional tools:**	
**Prognostic tool**	**Reason for exclusion from comparative analysis**
Charlotte large artery occlusion endovascular therapy outcome score (CLEOS) (1)	Serum glucose and CBV-index not available within GSR-ET database
Houston intra-arterial therapy (HIAT) (2)	Serum glucose not available within GSR-ET database
Houston intra-arterial therapy 2 (HIAT-2) (3)	Serum glucose not available within GSR-ET database
Italian Registry of Endovascular Stroke Treatment (IER)-START normogram(4)	End of procedure time not available within GSR-ET database
MR PREDICTS (5, 6)	Information on prior stroke, collateral score and serum glucose not available within GSR-ET database
Tor Vergata Stroke Score (TVSS) (7)	Serum glucose, collateral flow, Clot Burden Score not available within GSR-ET database
**Post-interventional tools:**	
**Prognostic tool**	**Reason for exclusion from comparative analysis**
GADIS (Gender, Age, Diabetes Mellitus History, Infarct Volume, and Sex) score (8)	Final infarct volume not available within GSR-ET database
Pitsburgh Outcomes after Stroke Thrombectomy (POTS) (9)	Final infarct volume not available within GSR-ET database
SNARL (10)	Subtype classification of symptomatic hemorrhage type not available within GSR-ET database

Abbreviations: GSR-ET: German Stroke Registry – Endovascular Treatment

Table S2: Subgroup analyses of AUC comparison of scoring instruments for outcome prognostication.

**Good outcome**		n	Admission NIHSS	PRE	THRIVE-EVT	CLEAR	24 hour NIHSS
	Total Cohort	6612	0.705	0.757*	0.751*	0.731	0.864
Age	<50	385	0.688	0.66	0.704	0.685	0.855
	50-75	2942	0.686	0.713*	0.699*	0.677	0.875
	>75**	3285	0.726	0.748*	0.73	0.707	0.863
Sex	Male**	3304	0.689	0.741*	0.736*	0.714	0.865
	Female**	3307	0.722	0.768*	0.76*	0.744	0.865
Time Window	<=6h	4591	0.705	0.756*	0.757*	0.731	0.864
	>6h	1432	0.735	0.761*	0.761*	0.756*	0.865
Stroke severity	NIHSS <10**	1859	0.562	0.652*	0.653*	0.611	0.798
	NIHSS 10-19	3606	0.613	0.719*	0.707*	0.662	0.858
	NIHSS >=20	1147	0.571	0.718*	0.652	0.686	0.836
ASPECTS	0-5**	530	0.784	0.818	0.806	0.809	0.867
	>5**	6082	0.695	0.755*	0.745	0.719	0.861
Combination Therapy	Bridging IVT	3341	0.7	0.754*	0.755*	0.73	0.863
	MT alone	3261	0.724	0.755*	0.749	0.74	0.863
Recanalization Status	Tici 2b-3	5607	0.705	0.759*	0.758*	0.734	0.852
	Tici 0-2a	920	0.763	0.78	0.778	0.791*	0.895

**Mortality**			Admission NIHSS	PRE	THRIVE-EVT	CLEAR	24 hour NIHSS
	Total Cohort	6612	0.682	0.717*	0.722*	0.705	0.814
Age	<50**	385	0.708	0.591	0.729	0.693	0.822
	50-75	2942	0.684	0.681	0.687	0.678	0.811
	>75	3285	0.672*	0.683*	0.672*	0.653	0.817
Sex	Male	3304	0.67	0.709	0.725*	0.7	0.81
	Female	3307	0.691	0.721*	0.716*	0.707*	0.817
Time Window	<=6h**	4591	0.683	0.722*	0.725*	0.707	0.831
	>6h**	1432	0.705	0.729	0.745*	0.732*	0.782
Stroke severity	NIHSS <10	1859	0.554	0.65*	0.67*	0.607	0.782
	NIHSS 10-19	3606	0.604	0.672*	0.672*	0.64	0.796
	NIHSS >=20**	1147	0.56	0.642*	0.643*	0.639*	0.755
ASPECTS	0-5	530	0.674	0.729*	0.727*	0.724*	0.763*
	>5	6082	0.676	0.718*	0.719*	0.696	0.816
Combination Therapy	Bridging IVT**	3341	0.687	0.74*	0.737*	0.715	0.837
	MT alone**	3261	0.685	0.69	0.709*	0.7	0.791
Recanalization Status	Tici 2b-3	5607	0.686	0.72	0.727*	0.71	0.814
	Tici 0-2a	920	0.679	0.695	0.73*	0.706	0.745*

Good outcome		n	MR PREDICTS @24H	BET	24 hour NIHSS
	Total Cohort	5594	0.889*	0.777	0.861
Age	<50	336	0.863*	0.759	0.862*
	50-75	2547	0.886*	0.801	0.871
	>75	2711	0.877*	0.786	0.858
Sex	Male	2778	0.891*	0.780	0.863
	Female	2815	0.887*	0.781	0.862
Time Window	<=6h	3915	0.888*	0.780	0.861
	>6h	1224	0.893*	0.769	0.863
Stroke severity	NIHSS <10**	1502	0.834*	0.690	0.787
	NIHSS 10-19	3100	0.883*	0.769	0.850
	NIHSS >=20	992	0.870*	0.750	0.836
ASPECTS	0-5	446	0.874*	0.755	0.857
	>5	4741	0.885*	0.772	0.855
Combination Therapy	Bridging IVT	2838	0.886*	0.776	0.859
	MT alone	2745	0.889*	0.777	0.861
Recanalization Status	Tici 2b-3**	5199	0.886*	0.767	0.856
	Tici 0-2a**	395	0.902*	0.821	0.877

Legend:

AUC of stated scoring instrument in stated (sub)group.

* significantly better AUC than non-coloured pre-/post-interventional Scoring instruments
Light green: Superiority in AUC comparison to all other instruments.
Dark green: Pre-/post-interventional scoring instrument of choice in the respective (sub)group resulting from AUC comparison.
Yellow: Not underperforming compared to instrument of choice, yet not superior to non-colored scores.

** Subgroup with significantly different AUC to other group in subgroups in comparison of independent ROC curves of instrument of choice for this oucome (good functional outcome: PRE, mortality: THRIVE-EVT, post-interventional: MR PREDICTS@24H)

Table S3: Results of sensitivity analysis. AUCs of scoring tools for prediction of desirable outcome (return to pre-stroke mRS) at 90-day follow-up in patients with pre-stroke mRS >2.

	**AUC**	**Lower Confidence Limit**	**Upper Confidence Limit**
**Complete cases:**			
Admission NIHSS	0.623	0.584	0.663
PRE	0.678	0.642	0.714
THRIVE-EVT	0.646	0.607	0.686
CLEAR	0.652	0.615	0.688
BET	0.720	0.677	0.762
MR PREDICTS@24H	0.846	0.815	0.878
NIHSS 24 hours after EVT	0.800	0.764	0.836
**Multiple imputation:**			
Admission NIHSS	0.653	0.626	0.681
PRE	0.702	0.677	0.728
THRIVE-EVT	0.683	0.656	0.710
CLEAR	0.674	0.648	0.701
BET	0.716	0.691	0.742
MR PREDICTS@24H	0.865	0.846	0.884
NIHSS 24 hours after EVT	0.819	0.797	0.841

Abbreviations: AUC: Area under the receiver operating characteristic curve; NIHSS: National Institutes of Health Stroke Scale; PRE: Pittsburgh Response to Endovascular therapy score, THRIVE-EVT: Totaled Health Risks in Vascular Events score – Endovascular therapy; CLEAR: Computed Tomography for Late Endovascular Reperfusion score; BET: Bronx Endovascular Thrombectomy score; EVT: endovascular therapy.

Table S4: Results of sensitivity analysis. AUCs of scoring tools for prediction of 90-day mortality in patients with pre-stroke mRS >2.

	AUC	Lower Confidence Limit	Upper Confidence Limit
**Complete cases:**			
Admission NIHSS	0.635	0.602	0.668
PRE	0.669	0.637	0.701
THRIVE-EVT	0.658	0.625	0.691
CLEAR	0.648	0.617	0.680
BET	0.692	0.655	0.729
MR PREDICTS@24H	0.805	0.774	0.836
NIHSS 24 hours after EVT	0.778	0.745	0.811
**Multiple imputation:**			
Admission NIHSS	0.666	0.641	0.692
PRE	0.692	0.666	0.717
THRIVE-EVT	0.685	0.660	0.710
CLEAR	0.674	0.649	0.699
BET	0.720	0.694	0.745
MR PREDICTS@24H	0.835	0.815	0.856
NIHSS 24 hours after EVT	0.816	0.794	0.837

Abbreviations: AUC: Area under the receiver operating characteristic curve; NIHSS: National Institutes of Health Stroke Scale; PRE: Pittsburgh Response to Endovascular therapy score, THRIVE-EVT: Totaled Health Risks in Vascular Events score – Endovascular therapy; CLEAR: Computed Tomography for Late Endovascular Reperfusion score; BET: Bronx Endovascular Thrombectomy score; EVT: endovascular therapy.

Table S5: Results of sensitivity analysis by multiple imputation.

Multiple imputation was performed using R (Version 4.3.1) and R-package mice (11). Predictive mean modelling was used to impute all missing variables needed for score calculation and outcomes. Imputation was repeated thirty times, resulting in thirty complete datasets with no missing values. AUROCs were calculated for all scores and endpoints for each imputed dataset, results were pooled using Rubin's rules. The resulting AUCs were qualitatively compared to the main analyses to assess a potential selection bias in the complete case dataset.

	**AUC**	**Lower Confidence Limit**	**Upper Confidence Limit**
**Pre-interventional scores**			
Good functional outcome (mRS 0-2) at 90-day follow-up			
Admission NIHSS	0.700	0.690	0.711
PRE	0.750	0.741	0.760
THRIVE-EVT	0.740	0.730	0.750
CLEAR	0.724	0.714	0.734
90-day mortality			
Admission NIHSS	0.682	0.669	0.694
PRE	0.715	0.703	0.727
THRIVE-EVT	0.719	0.707	0.731
CLEAR	0.704	0.692	0.716
**Post-interventional scores**			
Good functional outcome (mRS 0-2) at 90-day follow-up			
BET	0.781	0.771	0.791
MR PREDICTS@24H	0.885	0.877	0.892
NIHSS 24 hours after EVT	0.859	0.851	0.868
90-day mortality			
BET	0.723	0.711	0.736
MR PREDICTS@24H	0.843	0.834	0.853
NIHSS 24 hours after EVT	0.813	0.803	0.824

Abbreviations: AUC: Area under the receiver operating characteristic curve; NIHSS: National Institutes of Health Stroke Scale; PRE: Pittsburgh Response to Endovascular therapy score, THRIVE-EVT: Totaled Health Risks in Vascular Events score – Endovascular therapy; CLEAR: Computed Tomography for Late Endovascular Reperfusion score; BET: Bronx Endovascular Thrombectomy score; EVT: endovascular therapy.

Table S6. Comparative AUC analyses of LVO outcome prediction by scoring tools including pre-interventional information and NIHSS in patients discharged from treating hospital.

Predictive instrument	AUC	95% CI
Outcome parameter: Good functional outcome (mRS 0-2) at 90-day follow-up (n=5,243)		
Admission NIHSS	0.669	0.656–0.681
PRE	**0.727***	0.715–0.739
THRIVE-EVT	**0.719***	0.706–0.731
CLEAR	0.697	0.684–0.709
NIHSS 24 hours after EVT	**0.826****	0.816–0.836
Outcome parameter: 90-day mortality (n=5,243)		
Admission NIHSS	0.623	0.609–0.636
PRE	**0.685***	0.672–0.697
THRIVE-EVT	**0.691***	0.678–0.703
CLEAR	0.66	0.647–0.673
NIHSS 24 hours after EVT	**0.706***	0.693–0.718

*Abbreviations: AUC: Area under the receiver operating characteristic curve; NIHSS: National Institutes of Health Stroke Scale; PRE: Pittsburgh Response to Endovascular therapy score, THRIVE-EVT: Totaled Health Risks in Vascular Events score – Endovascular therapy; CLEAR: Computed Tomography for Late Endovascular Reperfusion score; EVT: endovascular therapy. *: significantly higher AUC than non-bold scoring tools. **: significantly higher AUC than all other scoring tools.*

Table S7. Comparative AUC analyses of LVO outcome prediction by scoring tools inlcuding post-interventional information and NIHSS in patients discharged from treating hospital.

Predictive instrument	AUC	95% CI
Outcome parameter: Good functional outcome (mRS 0-2) at 90-day follow-up (n=4,522)		
BET	0.744	0.731-0.756
MR PREDICTS@24H	**0.859***	0.849-0.869
NIHSS 24 hours after EVT	0.824	0.813-0.835
Outcome parameter: 90-day mortality (n=4,522)		
BET	0.643	0.629-0.657
MR PREDICTS@24H	**0.760***	0.748-0.773
NIHSS 24 hours after EVT	0.706	0.692-0.719

*Abbreviations: AUC: Area under the receiver operating characteristic curve; NIHSS: National Institutes of Health Stroke Scale; BET: Bronx Endovascular Thrombectomy score; EVT: endovascular therapy. *: significantly higher AUC than non-bold scoring tools.*

Figure S1:

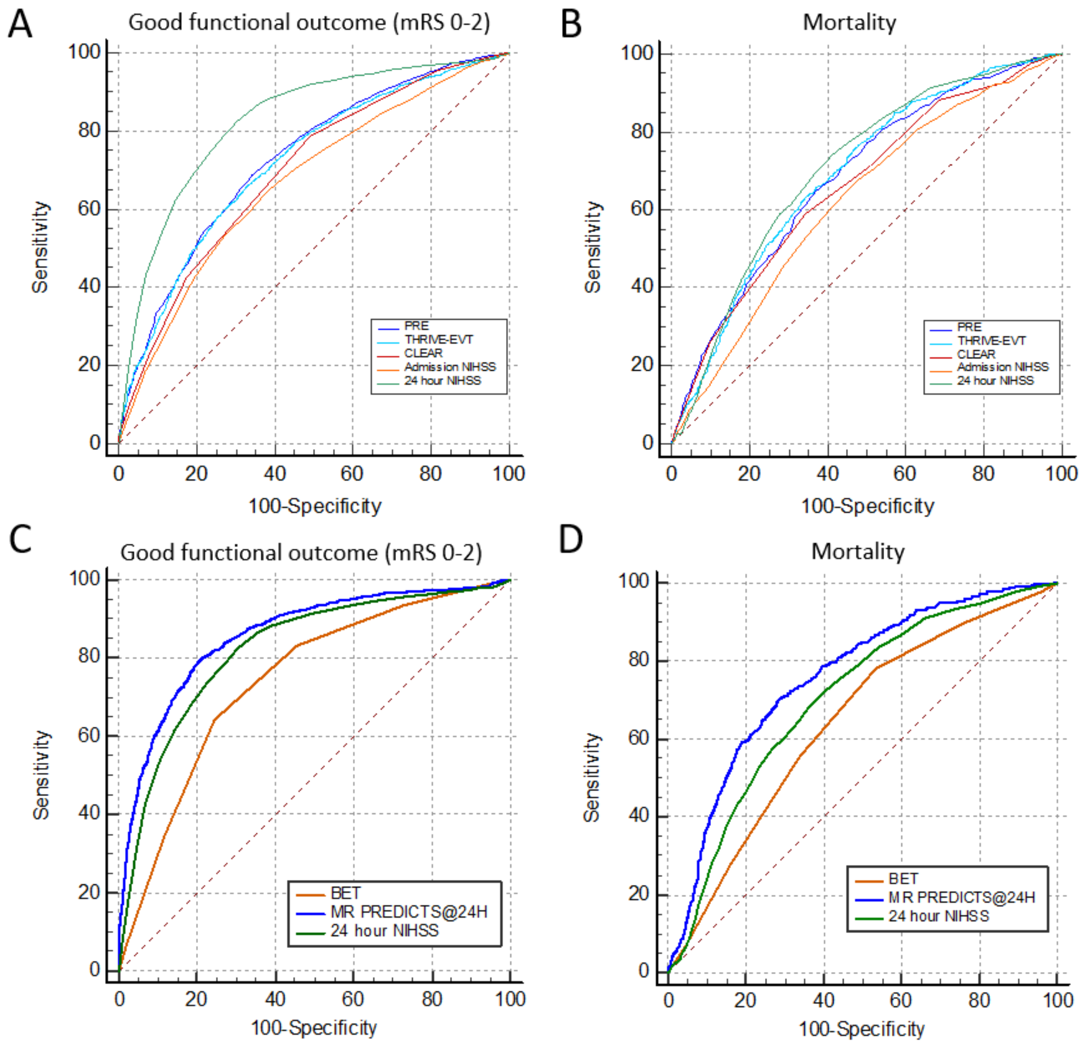

Figure S1: Subgroups analysis in patients discharged from treating hospital: A+B: AUC comparison of pre-interventional prognostic tools with admission NIHSS and NIHSS 24 hours after EVT predicting good functional outcome and mortality 90 days after EVT. C+D: AUC comparison of post-interventional prognostic tool with admission NIHSS and NIHSS 24 hours after EVT predicting good functional outcome and mortality 90 days after EVT. Abbreviations: mRS: modified Rankin scale score, NIHSS: National Institute of Health Stroke Scale; PRE: Pittsburgh Response to Endovascular therapy score, THRIVE-EVT: Totaled Health Risks in Vascular Events score – Endovascular therapy; CLEAR: Computed Tomography for Late Endovascular Reperfusion score, BET: Bronx Endovascular Thrombectomy score.

Figure S2: Model calibration analyses of pre-interventional scoring tools.

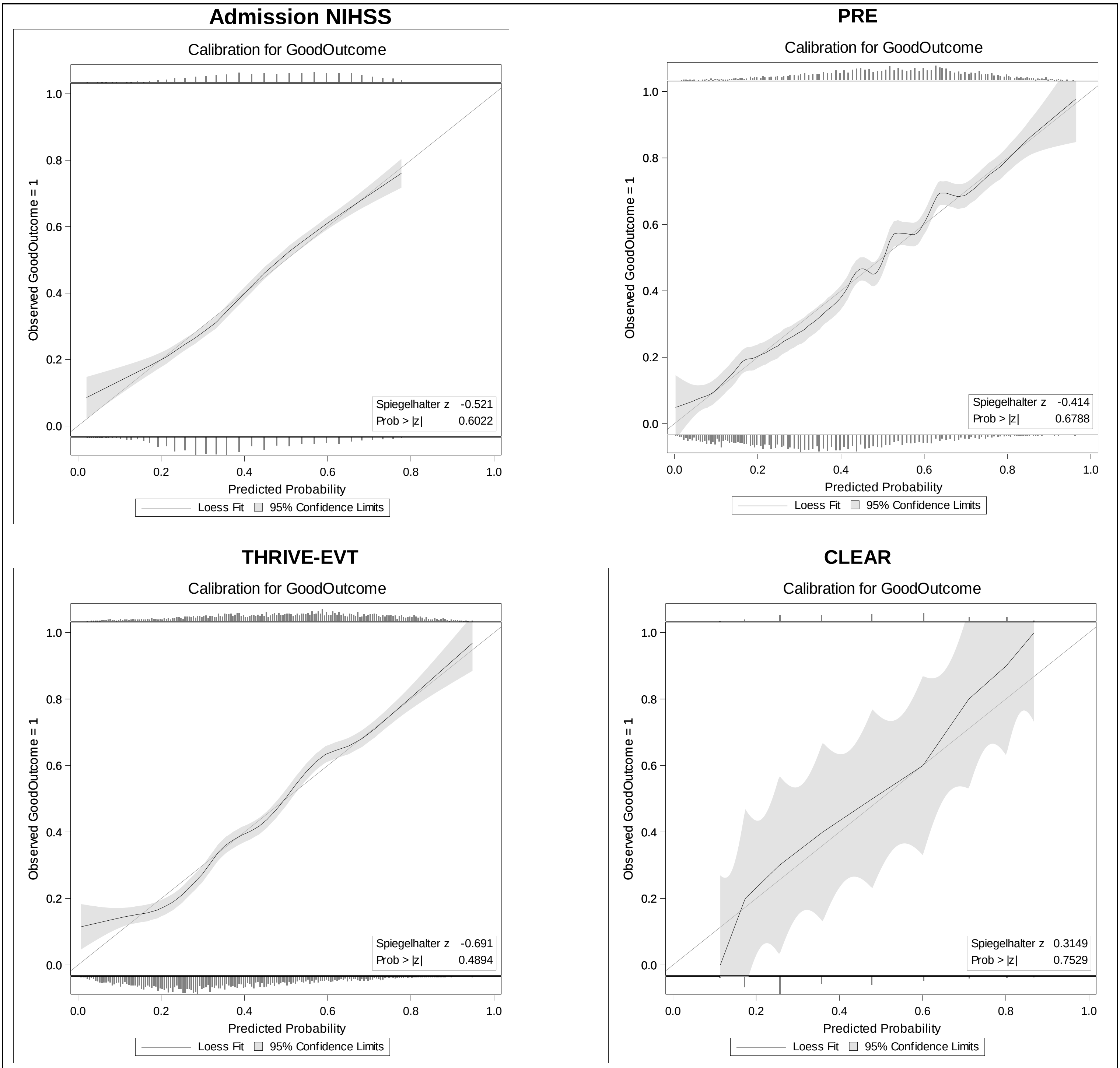

Score	Error Rate	AUC	AIC	AICC	BIC	SC	R-Square	Max-Rescaled R-Square	Brier Score
Admission NIHSS	0.3315	0.705224	8145.023	8145.025	8158.617	8158.617	0.116903	0.157509	0.212646
PRE	0.2954	0.757223	7596.885	7596.887	7610.479	7610.479	0.18716	0.252169	0.19543
THRIVE-EVT	0.2989	0.750853	7698.247	7698.249	7711.84	7711.84	0.174603	0.235251	0.197749
CLEAR	0.3164	0.731291	7870.636	7870.637	7884.229	7884.229	0.1528	0.205875	0.204468

Model calibration was assessed using calibration plots. A locally estimated scatterplot smoothing (LOESS) spline was fitted including 95%-confidence bands. Large deviations from the diagonal are a sign for poor calibration of predicted versus observed event probabilities. Additionally, Brier scores and Spiegelhalter tests were performed. Larger Brier scores and significant p-values are also a sign for inadequate calibration. Abbreviations: NIHSS: National Institute of Health Stroke Scale; PRE: Pittsburgh Response to Endovascular therapy score, THRIVE-EVT: Totaled Health Risks in Vascular Events score – Endovascular therapy; CLEAR: Computed Tomography for Late Endovascular Reperfusion score.

Figure S3: Model calibration analyses of post-interventional scoring tools.

BET

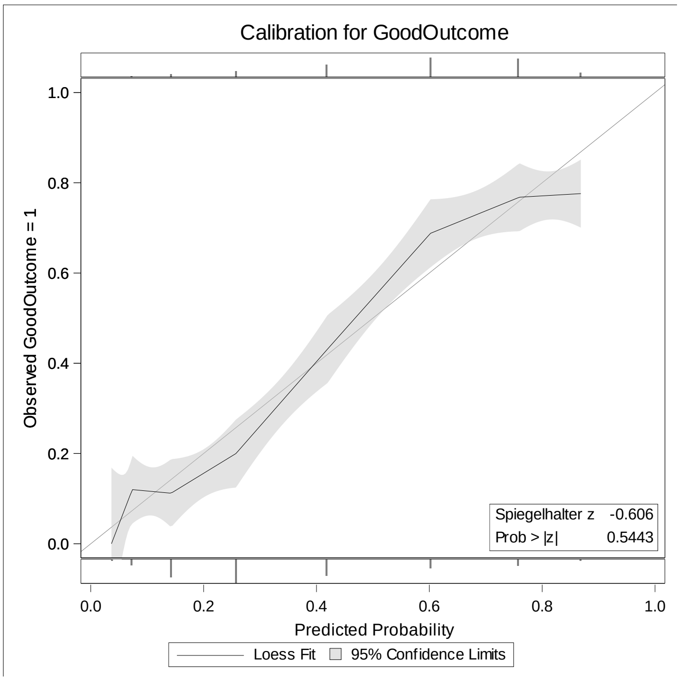

MR PREDICTS@24H

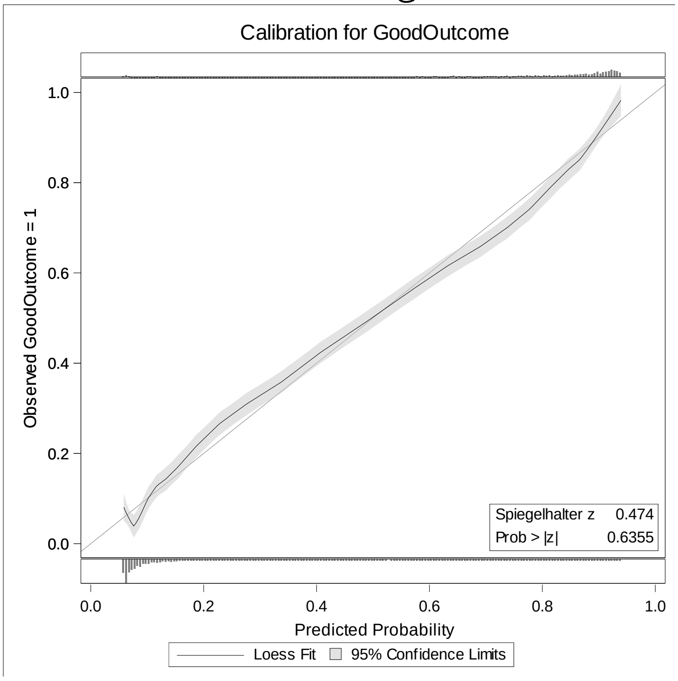

24-hour NIHSS

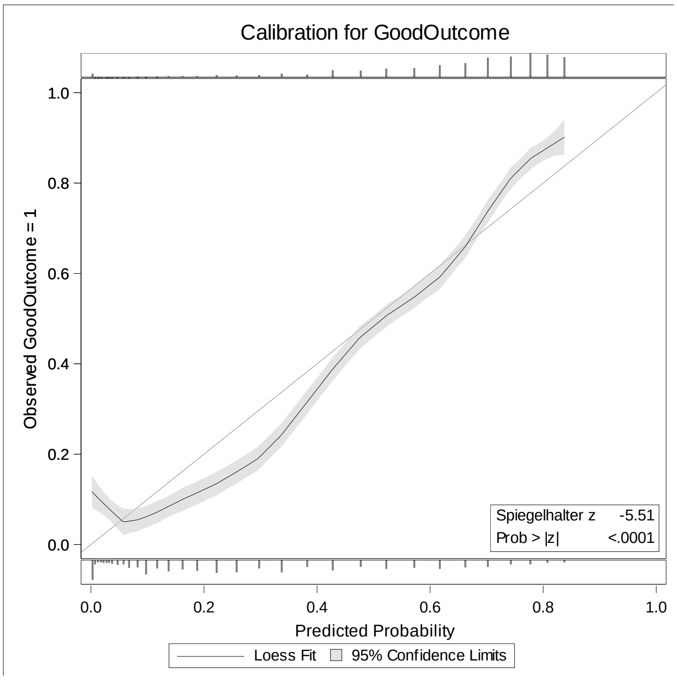

Score	Error Rate	AUC	AIC	AICC	BIC	SC	R-Square	Max-Rescaled R-Square	Brier Score
BET	0.2670	0.776625	6259.047	6259.049	6272.298	6272.298	0.219408	0.294126	0.188219
MR PRE DICTS@24H	0.1796	0.888443	4638.568	4638.57	4651.819	4651.819	0.416393	0.558193	0.130836
24 hour NIHSS	0.2096	0.860365	5482.593	5482.595	5495.844	5495.844	0.320944	0.43024	0.149923

Model calibration was assessed using calibration plots. A locally estimated scatterplot smoothing (LOESS) spline was fitted including 95%-confidence bands. Large deviations from the diagonal are a sign for poor calibration of predicted versus observed event probabilities. Additionally, Brier scores and Spiegelhalter tests were performed. Larger Brier scores and significant p-values are also a sign for inadequate calibration. Abbreviations: NIHSS: National Institute of Health Stroke Scale; BET: Bronx Endovascular Thrombectomy score.

Literature Cited

1. Karamchandani RR, Satyanarayana S, Yang H, Defilipp G, Strong D, Rhoten JB et al. The Charlotte large artery occlusion endovascular therapy outcome score predicts outcome after basilar artery thrombectomy. J Neuroimaging 2022; 32(5):860–5.

2. Hallevi H, Barreto AD, Liebeskind DS, Morales MM, Martin-Schild SB, Abraham AT et al. Identifying patients at high risk for poor outcome after intra-arterial therapy for acute ischemic stroke. Stroke 2009; 40(5):1780–5.

3. Sarraj A, Albright K, Barreto AD, Boehme AK, Sitton CW, Choi J et al. Optimizing prediction scores for poor outcome after intra-arterial therapy in anterior circulation acute ischemic stroke. Stroke 2013; 44(12):3324–30.

4. Cappellari M, Mangiafico S, Saia V, Pracucci G, Nappini S, Nencini P et al. IER-START nomogram for prediction of three-month unfavorable outcome after thrombectomy for stroke. Int J Stroke 2020; 15(4):412–20.

5. Venema E, Mulder MJHL, Roozenbeek B, Broderick JP, Yeatts SD, Khatri P et al. Selection of patients for intra-arterial treatment for acute ischaemic stroke: development and validation of a clinical decision tool in two randomised trials. BMJ 2017; 357:j1710.

6. Venema E, Roozenbeek B, Mulder MJHL, Brown S, Majoie CBLM, Steyerberg EW et al. Prediction of Outcome and Endovascular Treatment Benefit: Validation and Update of the MR PREDICTS Decision Tool. Stroke 2021; 52(9):2764–72.

7. Sallustio F, Toschi N, Mascolo AP, Marrama F, Morosetti D, Da Ros V et al. Selection of anterior circulation acute stroke patients for mechanical thrombectomy. J Neurol 2019; 266(11):2620–8.

8. O'Connor KP, Hathidara MY, Danala G, Xu C, McCoy TM, Sidorov EV et al. Predicting Clinical Outcome After Mechanical Thrombectomy: The GADIS (Gender, Age, Diabetes Mellitus History, Infarct Volume, and Current Smoker corrected) Score. World Neurosurg 2020; 134:e1130-e1142.

9. Rangaraju S, Liggins JTP, Aghaebrahim A, Streib C, Sun C-H, Gupta R et al. Pittsburgh outcomes after stroke thrombectomy score predicts outcomes after endovascular therapy for anterior circulation large vessel occlusions. Stroke 2014; 45(8):2298–304.

10. Prabhakaran S, Jovin TG, Tayal AH, Hussain MS, Nguyen TN, Sheth KN et al. Posttreatment variables improve outcome prediction after intra-arterial therapy for acute ischemic stroke. Cerebrovasc Dis 2014; 37(5):356–63.

11. Stef van Buuren, Karin Groothuis-Oudshoorn. mice: Multivariate Imputation by Chained Equations in R.
